# Bifunctional Fluorescent/Raman Nanoprobe for the Early Detection of Amyloid

**DOI:** 10.1038/s41598-019-43288-2

**Published:** 2019-06-11

**Authors:** Yang Xia, Parasuraman Padmanabhan, Sreelatha Sarangapani, Balázs Gulyás, Murukeshan Vadakke Matham

**Affiliations:** 1 School of Mechanical and Aerospace Engineering, Center for Optical and Laser Engineering (COLE), Nanyang Technological University (NTU), Singapore, 639798 Singapore; 2 Lee Kong Chian School of Medicine, Nanyang Technological University (NTU), Singapore, 637553 Singapore

**Keywords:** Nanoparticles, Biosensors

## Abstract

One of the pathological hallmarks of Alzheimer’s disease (AD) is the abnormal aggregation of amyloid beta (Aβ) peptides. Therefore the detection of Aβ peptides and imaging of amyloid plaques are considered as promising diagnostic methods for AD. Here we report a bifunctional nanoprobe prepared by conjugating gold nanoparticles (AuNPs) with Rose Bengal (RB) dye. RB is chosen due to its unique Raman fingerprints and affinity with Aβ peptides. After the conjugation, Raman signals of RB were significantly enhanced due to the surface-enhanced Raman scattering (SERS) effect. Upon binding with Aβ42 peptides, a spectrum change was detected, and the magnitude of the spectrum changes can be correlated with the concentration of target peptides. The peptide/probe interaction also induced a remarkable enhancement in the probes’ fluorescence emission. This fluorescence enhancement was further utilized to image amyloid plaques in the brain slices from transgenic mice. In this study, the RB-AuNPs were used for both SERS-based detection of Aβ42 peptides and fluorescence-based imaging of amyloid plaques. Compared to monofunctional probes, the multifunctional probe is capable to provide more comprehensive pathophysiological information, and therefore, the implementation of such multifunctional amyloid probes is expected to help the investigation of amyloid aggregation and the early diagnosis of AD.

## Introduction

Neurodegenerative disorders are diseases caused by the death of neurons and malfunction of the neural circuit. They can severely damage the memory, skilled movements, emotional feelings, cognition and other capabilities of patients. One important characteristic of neurodegenerative disorders is that the risk of disease increases with age^1^. Therefore, with a progressive increase of elder people in the world population, neurodegenerative diseases are projected to become a severer health threat, especially in developed countries, in which the life expectancy is commonly greater than 80 years old^2^. The most common type of neurodegenerative disorder is Alzheimer’s disease (AD), which is responsible for 60% to 70% of the recorded dementia cases^3^. The exact pathological mechanism of AD is still not well studied, but the most widely accepted explanation is the Amyloid hypothesis. Amyloid plaques are the insoluble, extracellular and proteinaceous aggregates formed by misfolded proteins or peptides which are normally soluble^4^. Although amyloid can also be found in other body parts including skin, pancreases and tongue^5^, the brain amyloid is the most definitive histopathologic hallmark for AD. The amyloid plaque is formed by a certain group of peptides known as the amyloid beta (Aβ) peptides. These peptides are produced by proteolytic cleavage of a transmembrane protein called the amyloid precursor protein (APP). Aβ peptides have various lengths depending on the site of cleavage, but most Aβ peptides are those with 40 (Aβ40) or 42 (Aβ42) amino acids^6^. These Aβ monomeric peptides are highly unstable and have a great tendency to assemble into oligomers, and further develop into large insoluble plaques. While Aβ40 are more abundant, the Aβ42 peptides are considered as the major pathologic contributor due to its higher toxicity and faster fibrillation rate^7^. A series of toxic species is produced during the amyloid aggregation process and eventually, lead to the death of neurons. One thing to be emphasized is that the molecular onset of amyloid aggregation may start long before the appearance of detectable clinical changes. Therefore, the detection and imaging of amyloid peptides and fibrils are expected to be utilized for the early diagnosis of AD^8^.

Surface-enhanced Raman scattering (SERS) is a phenomenon which shows dramatically enhanced (up to 10^10^ factors) Raman scattering when molecules are present in the close vicinity of nanostructures consisting of certain noble metal, transition metal, or semiconductors^9^. The origin of the SERS effect is generally believed to be a combined effect from the electromagnetic field (EM) enhancement and the chemical enhancement. EM field enhancement exists due to the locally-enhanced electromagnetic field on the surface of metal nanostructures, which consequently increases the excitation intensities of the Raman scattering. Chemical enhancement is attributed to the charge transfer between the metal substrate and the adsorbed molecules. The charge transfer is a result of a Raman resonance in which the electronic states of the adsorbate are increased, shifted and broadened. This resonance enables the charge transfer excitation between the highest occupied molecular orbital (HOMO) and the lowest unoccupied molecular orbital (LUMO) of the adsorbate at an energy half of the intrinsic intramolecular excitations. The effect of chemical enhancement largely depends on the type of adsorbed molecules. On the other hand, the EM field enhancement is universal for all molecule types^10,11^. Over the past decade, SERS spectroscopy has been utilized as a powerful analytical tool in chemistry, material science and biomedical research. It is capable of revealing the conformational changes and structural differences between different molecules with high sensitivity. However, for protein detection, some obstacles still exist. First of all, significant SERS effect is generally restricted within 2 nm around the surface of nanostructures, therefore hinders the direct detection of large biomolecules such as protein^12,13^. In addition, the intrinsic molecular and conformational complexity of proteins often generates complicated spectral patterns, which makes it very difficult to identify characteristic Raman fingerprints. The protein-probes interactions may vary due to the different surrounding medium, which leads to a low batch-to-batch reproducibility^12,14^. To overcome the aforementioned limitations, an indirect-SERS strategy has been proposed: instead of interrogating the Raman signals from the proteins, an additional Raman reporter with large Raman cross-section can be attached to the nanostructures and the reporter is allowed to react with the target protein in order to generate detectable changes in the Raman spectra^15,16^. This type of SERS probes has been successfully applied to detect DNA, protein and other bio-molecules^17^.

Apart from detection/sensing applications, SERS probes have also been used for bio-imaging applications such as cell labeling, tissue diagnosis and *in-vivo* imaging on small animal models^18^. While SERS based Raman imaging shows great advantages in terms of sensitivity and multiplexing capability, the long image acquisition time and the requirement of post-processing have limited the temporal resolution of Raman imaging^19^. To overcome this speed limit and also extend the capability of SERS probes, other imaging moieties, including fluorescent imaging^19–21^, MRI^22^ and X-ray CT^23^, have been integrated with SERS. These modality integrations generated a requirement of multifunctional SERS probes. Among these multifunctional SERS probes, fluorescent SERS (F-SERS) probe is the most well-studied probe. One reason is that fluorescence imaging is an intuitive method and can achieve real-time image acquisition, which makes it an ideal complementary imaging modality for Raman imaging. Another advantage of F-SERS probes is that many fluorescent dyes such as fluorescein isothiocyanate (FITC)^20^ and Rhodamine B derivatives^24^ can be readily used as Raman reporters when they are conjugated with nanostructures, which makes the preparation of F-SERS probes much facile.

In this presented work, we report a bifunctional gold nanoparticles (AuNPs) for SERS based detection of Aβ42 peptides and fluorescence imaging of amyloid plaques. The AuNPs were functionalized by covalent conjugation with Rose Bengal (4,5,6,7-tetrachloro-2′,4′,5′,7′-tetraiodofluorescein). Rose Bengal (RB) is a fluorescein derivative that has been widely used as a tumor-targeting dye for oral cancer diagnosis^25,26^. By conjugating with gold nanoparticles, the unique SERS spectrum of RB has been exploited for Raman imaging^27^. Interestingly, RB also shows a strong affinity to Aβ peptides and therefore has been used as inhibitors for Aβ aggregation^28,29^. Inspired by these phenomena, we tried to conjugate RB with spherical, amine-functionalized AuNPs to generate RB-AuNPs complex. The RB-AuNPs exhibited a significant change of the SERS spectrum when mixed with Aβ42 peptides solutions. In addition, the fluorescence emission of the RB-AuNPs was considerably enhanced with the presence of Aβ42 peptides. This behavior is similar to that of Thioflavin T (ThT), a commonly used amyloid dye^30^. Therefore, we further tested the feasibility of using RB-AuNPs for fluorescence imaging of amyloid plaques in mouse brain tissues. These brain tissues were harvested from transgenic mice that can express human amyloid peptides. These results suggest that the conjugated RB-AuNPs can be used as a bifunctional probe for amyloid detection, and as a diagnostic tool for the onset of AD.

## Results

### Preparation and characterization of RB-AuNPs

**Figure 1:**
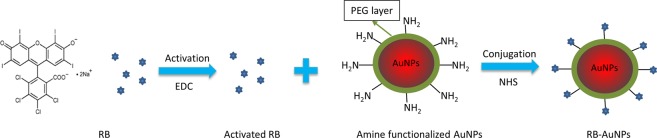
Scheme for the EDC/NHS facilitated conjugation for RB-AuNPs. The Carboxylic groups in the RB molecules are first activated by EDC and then conjugated with the amine groups on the surface of the AuNPs, in the presence of NHS as the catalyst.

**Figure 2:**
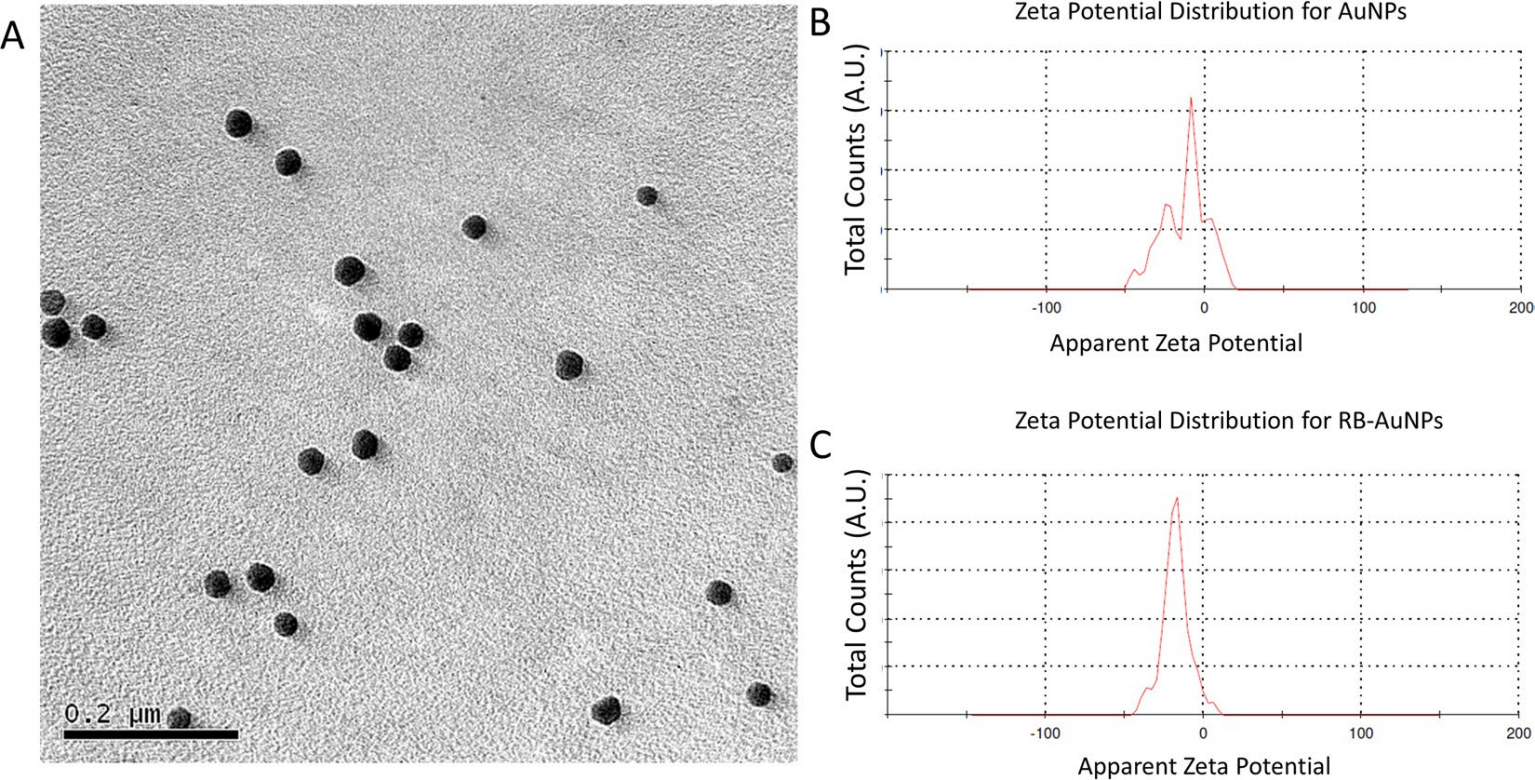
(**A**) TEM images of the RB-AuNPs, (**B**,**C**) Zeta potential distribution for the un-functionalized AuNPs (upper) and RB-AuNPs (lower).

**Figure 3:**
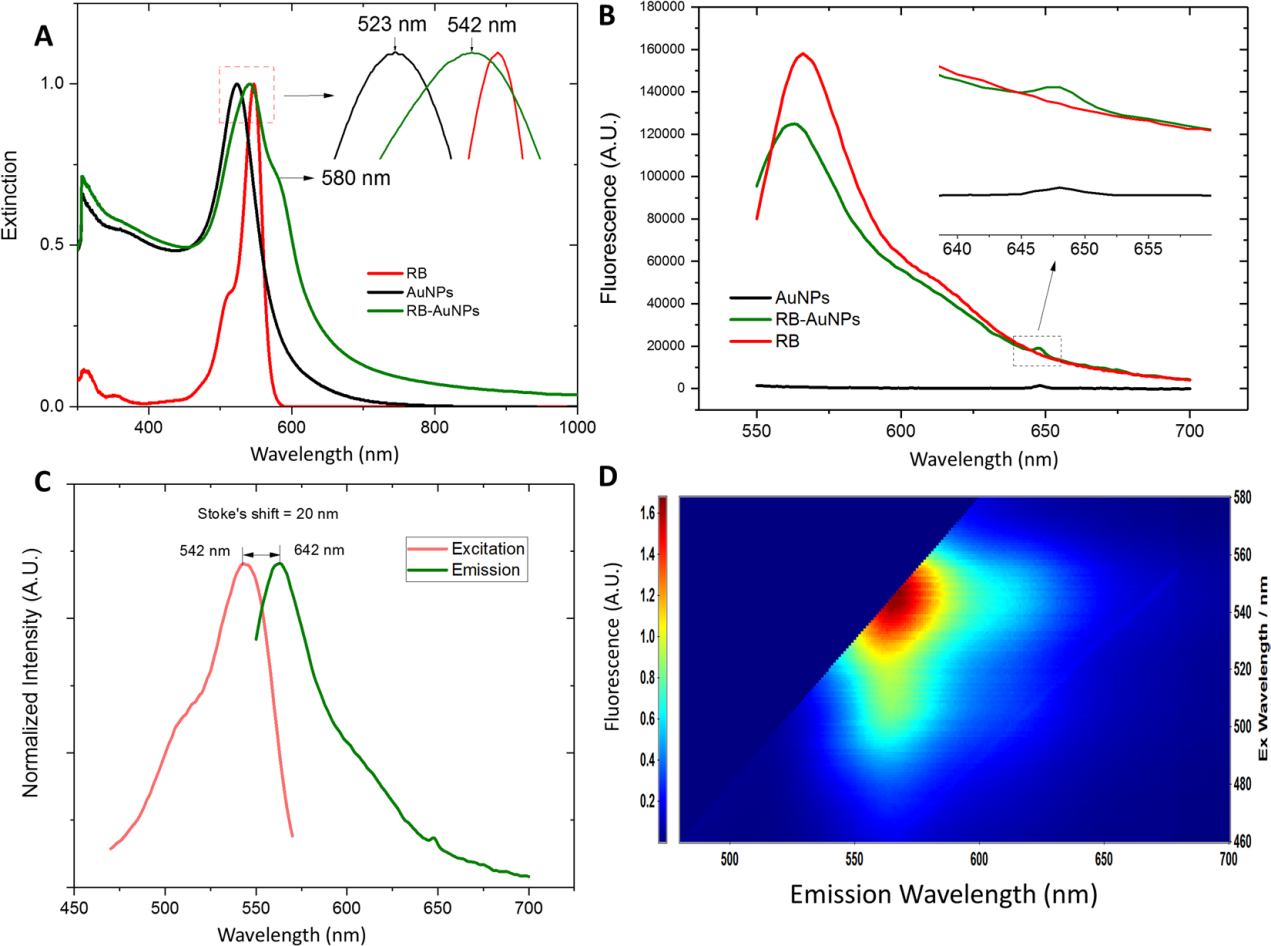
Optical characterizations of the RB-AuNPs. (**A**) Extinction spectra of RB, AuNPs and RB-AuNPs, (**B**) Emission spectra of RB, AuNPs and RB-AuNPs under 530 nm excitation, (**C**) excitation-emission spectrum of RB-AuNPs, the excitation spectrum was obtained at a fixed emission of 590 nm, and (**D**) Emission map of RB-AuNPs, plotted by setting the x-axis as emission wavelength and y-axis as excitation wavelength. The color represents the relative peak intensity of emission.

As illustrated by Fig. 1, the RB molecules were conjugated to the spherical AuNPs by crosslinking the carboxylic groups of RB with the surface amine groups of AuNPs. These amine groups come from the precoated amine-functionalized Poly(ethylene glycol) (PEG) layer. Although non-spherical AuNPs (nanorods, nano-triangles and nano-stars) may produce stronger SERS effect than spherical AuNPs, they also exhibit higher photo-toxicity due to their stronger absorption^31^. RB is capable of functioning as a photosensitizer for PDT^32^, by conjugating with gold nanorods, the synergy from the photodynamic effect of RB and the photothermal effect of gold nanorods leads to an enhanced, photo-induced cytotoxicity^33^. For therapeutic applications, this enhanced cytotoxicity is a desirable feature that increases the therapeutic efficiency. However, for diagnostic applications, the enhanced cytotoxicity is a disadvantage. In addition, at the same volume, the spherical nanoparticles have a higher mitigation rate than shape-anisotropic nanoparticles, which enables faster probe delivery in biological systems^34^. Taking the aforementioned two factors into consideration, we chose spherical AuNPs instead of non-spherical AuNPs. The TEM image of the functionalized RB-AuNPs is demonstrated in Fig. 2(A). It can be measured that the average diameter of the RB-AuNPs is 28 ± 3 nm. Due to the small size of conjugated RB moieties, the TEM images failed to show detectable changes in the core size of the nanoparticles before and after the conjugation. The RB-AuNPs maintained their good dispersity in aqueous solution. Differential light scattering measurement (DLS) also show that the hydrodynamic diameter of the nanoparticles only slightly increased (from 29.10 nm to 32.31 nm, Supplementary Fig. S1), suggesting that the original anti-aggregation surface coating of the AuNPs was not deteriorated by the RB conjugation. When transferred into the cell culture medium, a slight aggregation of RB-AuNPs particles occurred, revealed by the change of the nanoprobes’ UV-VIS spectra (Supplementary Fig. S2) Fig. 2(B,C) show the zeta potential distribution for the AuNPs before and after the RB conjugation. The apparent Zeta potential changed from −8.43 ± 3.68 mv to −17.2 ± 7.32 mv due to the conjugation of negatively charged RB molecules. Figure 3(A) shows the extinction spectra of the AuNPs, RB and RB-AuNPs. Compared to the original AuNPs, the extinction peak of the RB-AuNPs shows a redshift of 19 nm (523 to 542 nm) and an additional minor peak at the 580 nm wavelength. These changes could be attributed to the changes in surface environment^35^, which are considered as proof for the successful conjugation between RB and nanoparticles^33,36^. The emission spectra (Fig. 3B) of the AuNPs, RB and RB-AuNPs further prove the absorption of RB on the surface of AuNPs. The fluorescence of RB was not significantly quenched by the conjugation, and the weak photoluminescence (PL) of AuNPs at 648 nm was also retained after the conjugation. This PL of AuNPs is caused by the excited particle plasmon^37^. It is well known that AuNPs are fluorescence quencher and the quenching effect is distance-dependent: the quenching become more significant as the dye approaches the metallic surface of AuNPs^38^. Due to the random lengths and orientations of PEG molecules, the distances between RB and AuNPs surface vary throughout the vicinity of the AuNPs. Some of the RB molecules locate far enough from the particle and therefore retain their fluorescence. The excitation-emission spectrum of RB-AuNPs was shown in Fig. 3(C), the Stoke’s shift of RB-AuNPs was calculated to be 20 nm. The emission map of RB-AuNPs (Fig. 3D) indicates that the emission peaks is at 562 nm (542 nm excitation).

### Biocompatibility assay

**Figure 4:**
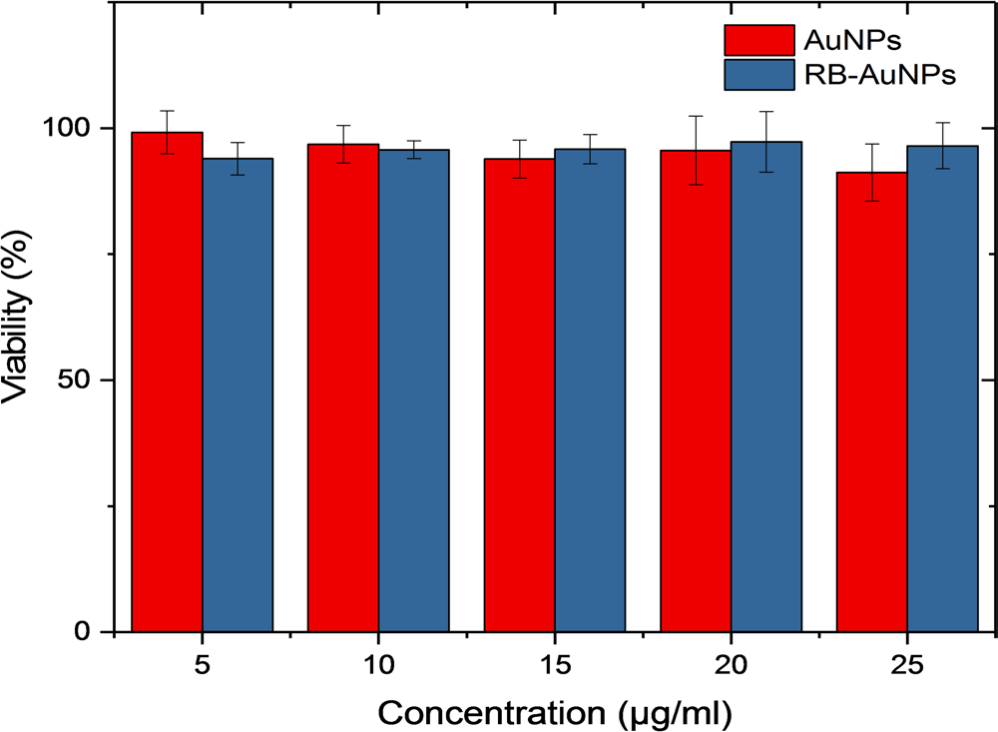
Cell viability assay of HEK-293 cell incubated 24 hours with AuNPs and RB-AuNPs. The viability was measured by MTT Assay. Three independent experiments were conducted for each concentration.

The cytotoxicity of the RB-AuNPs was measured by incubating different concentration of RB-AuNPs with HEK 293 cells. The results are listed in Fig. 4. AuNPs are generally considered as bio-compatible materials^39^. But RB molecules may produce toxic reactive oxygen species (ROS) under green light excitation, which can cause cell death^36,40^. From the MTT results, we can see that the functionalized RB-AuNPs are equally biocompatible as compared to the original AuNPs. Even at a relatively high incubation concentration (25 μg/ml), the cell viability is still greater than 95%. The cell morphology also remains intact when incubated with the RB-AuNPs (Supplementary Fig. S3). This high viability could be attributed to the limited light dose during the cell culture.

### SERS enhancement

**Figure 5:**
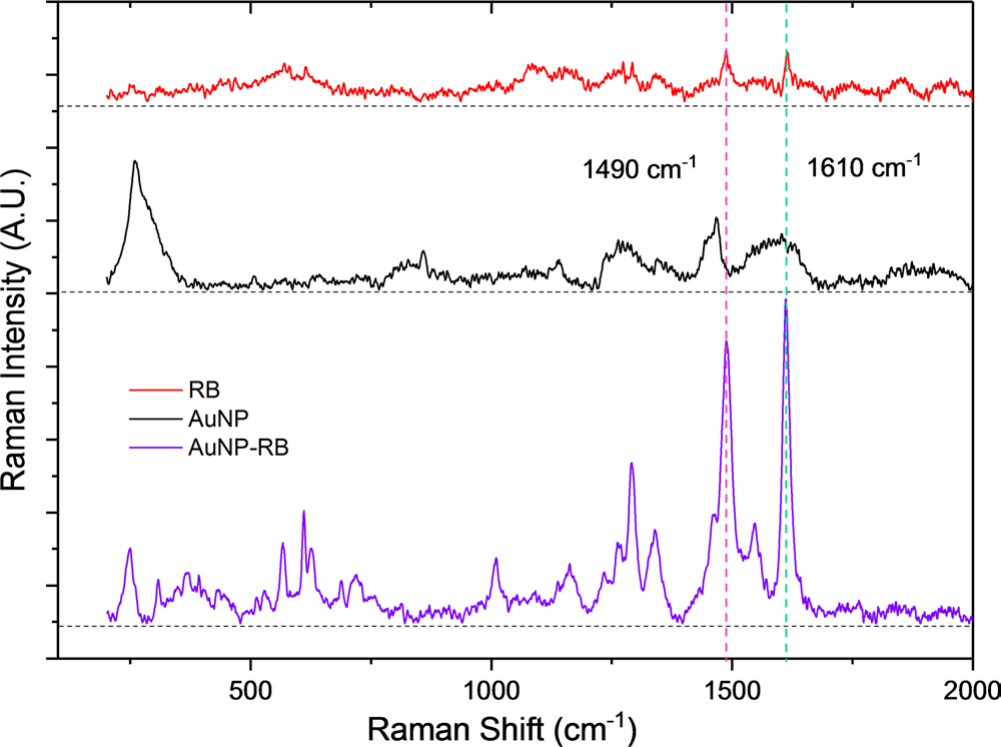
Raman spectra of RB, AuNPs and RB-AuNPs. The dashed line represent the baseline for each Raman spectrum. It is clear that the two peaks at 1490 cm^−1^ and 1610 cm^−1^ are most significantly enhanced after the conjugation.

In this work, the conjugated RB molecules served as both fluorescence and Raman reporter. Figure 5 shows the Raman spectra of RB, AuNPs and RB-AuNPs. The free RB molecules show very weak Raman signals and the peaks are overwhelmed by the background signals. The Un-conjugated AuNPs shows certain Raman peaks, these peaks are attributed to the pre-existed Polyethylene glycol-amine (PEG-NH_2_) coating on the surface of AuNPs and the strong peak at 200 to 400 cm^−1^ is caused by the metal-oxygen linking^41^. The Raman spectrum of RB-AuNPs shows a significant SERS effect, especially for the two major peaks located at 1490 and 1610 cm^−1^. Based on previously reported studies^42,43^, these two major peaks are attributed to the symmetric (for 1610 cm^−1^) and asymmetric (for 1490 cm^−1^) stretching of C=C bonds in the aromatic rings. The moderate peaks appeared between 550 cm^−1^ and 750 cm^−1^ are assigned to the vibrations of C-Cl bonds. It is well-known that the SERS effect will preferentially enhance these bands corresponds to the vibrations that cause polarizability changes perpendicular to the metal surface^43^. Therefore, the appearance of those moderate peaks indicating that the RB molecules are conjugated in such a way that the -COO^−^ groups of RB are pointing towards the surface of AuNPs.

### The interaction between Aβ42 and RB-AuNPs

**Figure 6:**
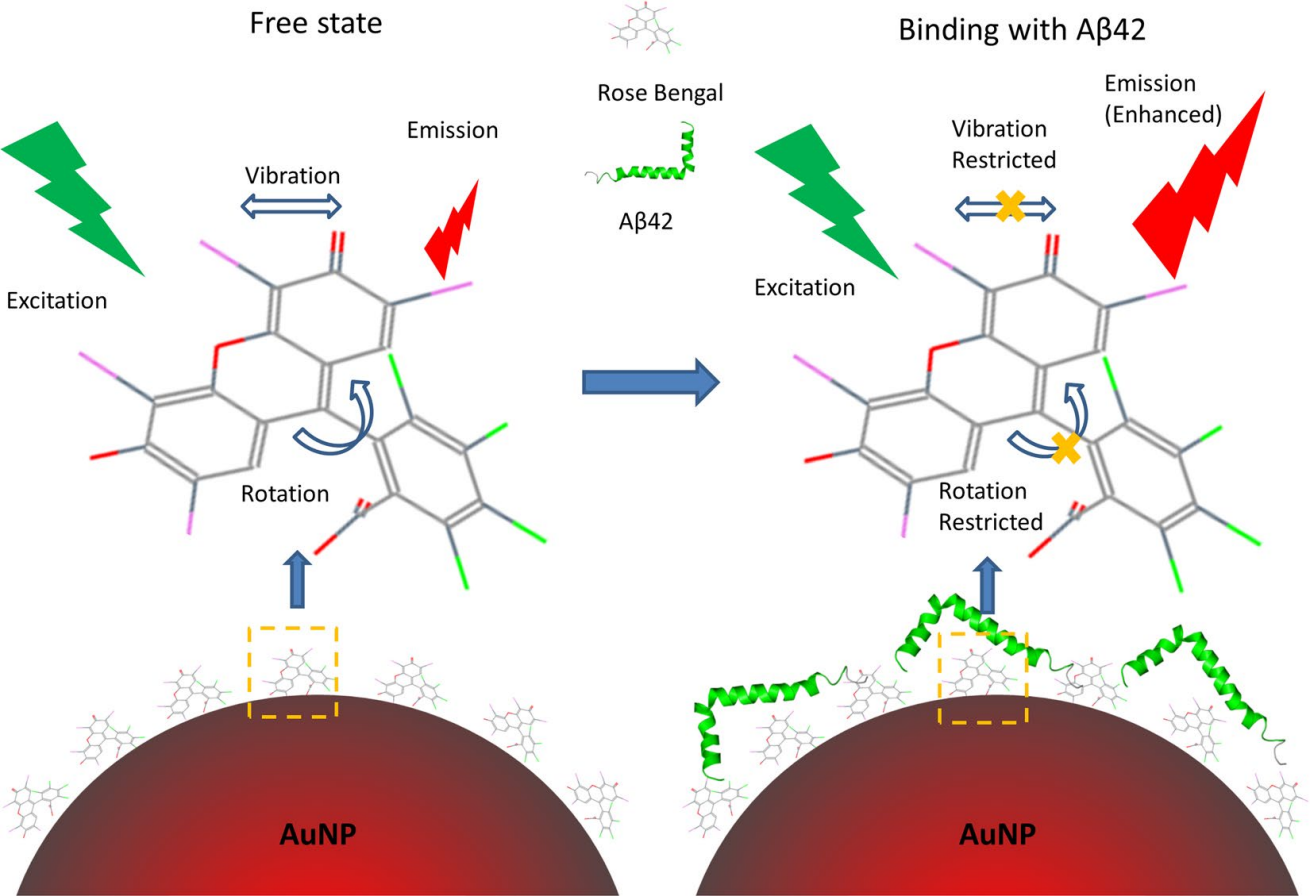
Schematic illustration of the RB-AuNPs & Aβ42 interaction. Prior to the peptides binding, the energy from the excitation photons can be released by the rotations and the vibrations of RB molecules. The vibrations and rotation are restricted after the peptides binding, leading to an enhanced fluorescence.

**Figure 7:**
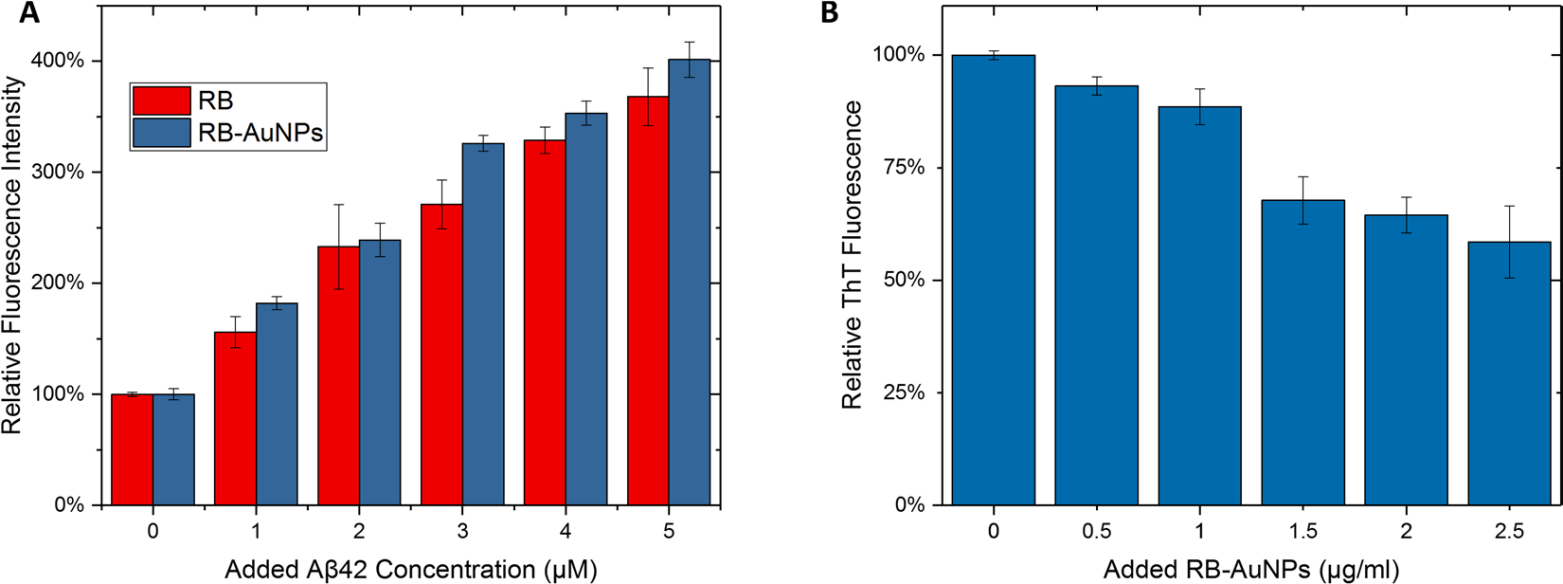
Fluorescence enhancement of RB-AuNPs and the effect of RB-AuNPs on ThT fluorescence. (**A**) Fluorescence enhancement of RB-AuNPs (1 μg/ml) and free RB (1 μM). The relative enhancement is calculated by comparing the fluorescence intensity (560/590 nm ex/em) before and after the addition of Aβ42 peptides. (**B**) The changes of ThT fluorescence (10 μM, mixed with 10 μM of Aβ42 peptides) after the addition of different concentrations of RB-AuNPs.

Previous reports have shown that the fluorescence of free RB molecules can be significantly enhanced upon mixing with Aβ42 peptides^29^. Several other fluorophores have shown similar fluorescence enhancement when mixed with peptides, and this enhancement phenomenon is considered as a proof of dye-peptide association^44–46^. The most widely accepted explanation for this fluorescence enhancement is an analogue of the ThT & Amyloid interaction mechanism (Fig. 6): At free state, the majority of the energy from the excitation photons can be released by the rotations and the vibrations of RB molecules, leading to a relatively low fluorescence emissions (RB’s Quantum Yield = 0.5% in PBS)^47^. Upon binding with Aβ42 peptides, the molecular rotations and vibrations are restricted, and therefore more energy has to be released by photon emission, causing an enhanced fluorescence^30,48^. From Fig. 7(A), it can be found that the fluorescence emission of both stand-alone RB (1 μM) and RB-AuNPs (1 μg/ml) were notably enhanced when mixed with Aβ42 peptides, and the enhancement increased with the amount of added Aβ42. It is interesting to see that the relative fluorescence enhancement of RB-AuNPs is actually slightly higher than that of free RB. This can be explained by the absorption of Aβ42 peptides on the surface of RB-AuNPs that lead to a higher interaction possibility between Aβ42 peptides and RB molecules. Figure 7(B) further indicates that the ThT and RB-AuNPs share a common binding pathway with Aβ42 peptides. The ThT fluorescence was remarkably reduced after the addition of RB-AuNPs.

### SERS detection of Aβ42 peptides

**Figure 8:**
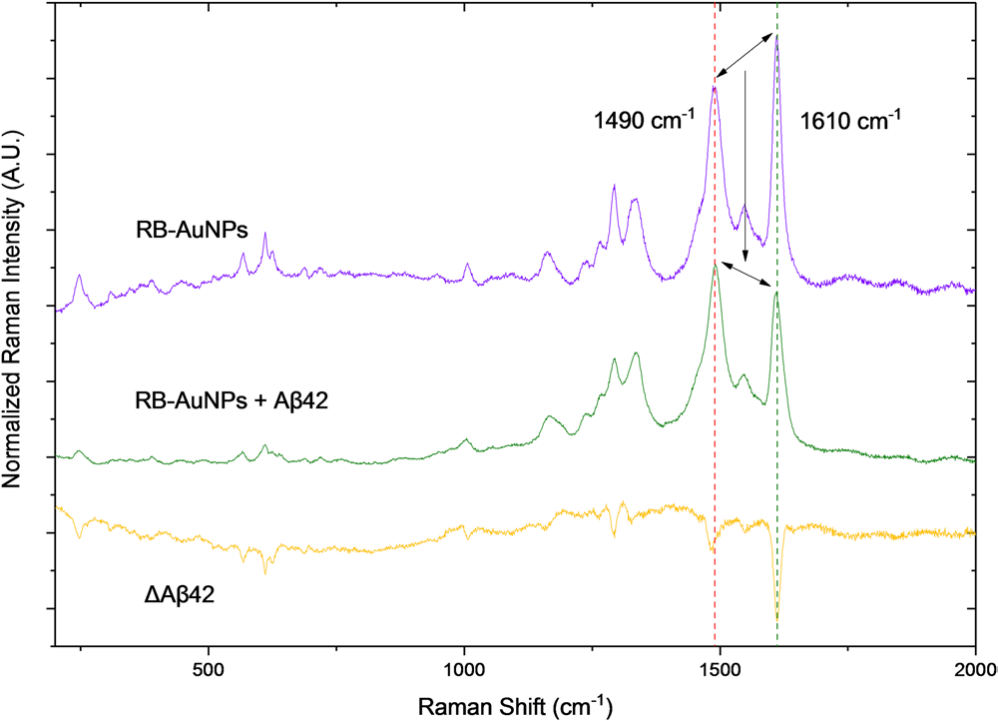
Aβ42 induced spectra changes of RB-AuNPs. The normalized Raman spectra of the RB-AuNPs before (violet) and after (green) the addition of Aβ42 peptides (2 μM) show a considerable change in the relative magnitude of the signals from the two major peaks. The orange curve is obtained by digital subtraction. of the (RB-AuNPs) spectrum from the (RB-AuNPs + Aβ42) spectrum. Prior to the subtraction, both spectra were normalized using the standard score method.

**Figure 9:**
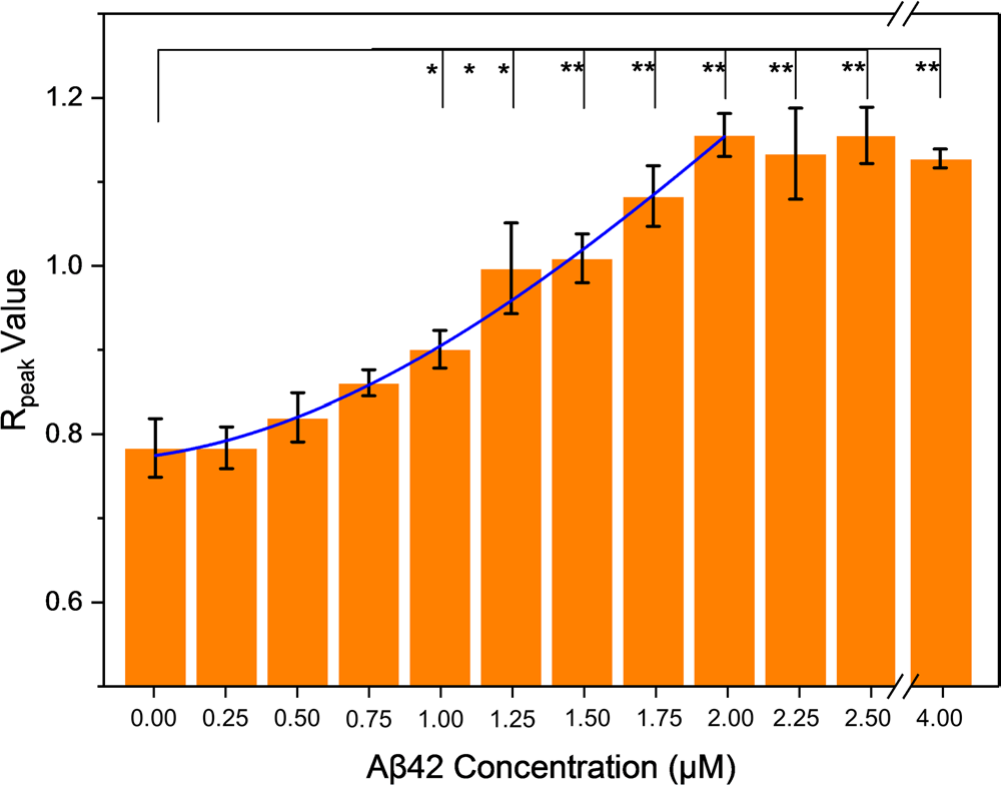
The relationship between R_peak_ and Aβ42 concentration. The Rpeak values are plotted against Aβ42 concentration (0, 0.25, 0.5, 0.75, 1, 1.25, 1.5, 1.75, 2, 2.25, 2.5 and 4 μM). The Rpeak value increases with the concertation of Aβ42. When the peptides concentration is greater than 2 μM, the peptide-to-particle ratio becomes high enough to saturate the surface of particles so that the increase of Rpeak value stops. The blue curve represents the Gaussian fitting in the range between 0 to 2 μM (R^2^ = 0.99532). Statistical comparisons were carried out base on the Student’s t-test. (*)P values < 0.05 or 0.01 (**) were considered significant.

The potential of the RB-AuNPs as SERS probes for amyloid detection was tested by exposing RB-AuNPs at different concentrations of Aβ42 peptides. The normalized Raman spectra of RB-AuNPs before and after the addition of 2 μM of Aβ42 peptides are demonstrated in Fig. 8. The spectrum changes caused by the Aβ42 peptides (ΔAβ42) can be fully revealed by digitally subtracting the original RB-AuNPs spectrum. The resulting ΔAβ42 spectrum shows that the most significant changes took place in the two major peaks in 1490 cm^−1^ and 1610 cm^−1^. These changes are associated with the mechanical deformation of RB aromatic rings sandwiched between the AuNPs and absorbed Aβ42 peptides^49,50^. The relative signal intensity in 1610 cm^−1^ underwent a much larger decrease compared to the signal in 1490 cm^−1^. Consequently, the relative peak strength between 1490 cm^−1^ and 1610 cm^−1^ was changed. The highest peak in the original RB-AuNPs spectrum locates at 1610 cm^−1^ and the peak at 1490 cm^−1^ is slightly lower. After exposing to Aβ42 peptides, the peak at 1490 cm^−1^ becomes the highest one. The ratio between the two peaks R_peak_ (intensity at 1490 cm^−1^/intensity at 1610 cm^−1^) changed from 0.780 to 1.194. To evaluate the quantitative correlation between the concentration of Aβ42 peptides and the spectral changes, Raman spectra were measured for RB-AuNPs mixed with different concentrations of Aβ42 peptides. For each concentration, 100 different spots were measured and the average R_peak_ values were plotted against the concentration of Aβ42 peptides (Fig. 9). The R_peak_ value and Aβ42 concertation show a clear positive correlation when the concentration of Aβ42 increased from 0 to 2 μM (r^2^ = 0.9739). When the Aβ42 concentration exceeded 2 μM, the surfaces of RB-AuNPs were saturated by the peptides and hence no further increase of R_peak_ value was observed. The selectivity of RB-AuNPs are tested by mixing another cerebral protein, Lipocalin prostaglandin D synthase (L-PGDS), with the nanoprobe and performed the same Raman spectroscopy. The results show that the changes in the ratio of major peaks are specifically associated with amyloid peptides. (Supplementary Fig. S5) It is also interesting to see that the changes of R_peak_ value are detectable even in mouse brain slices that were stained with RB-AuNPs (Supplementary Fig. S6), suggesting a potential amyloid mapping strategy based on R_peak_ values.

### Fluorescence imaging of amyloid plaques in transgenic mouse brain slices

**Figure 10:**
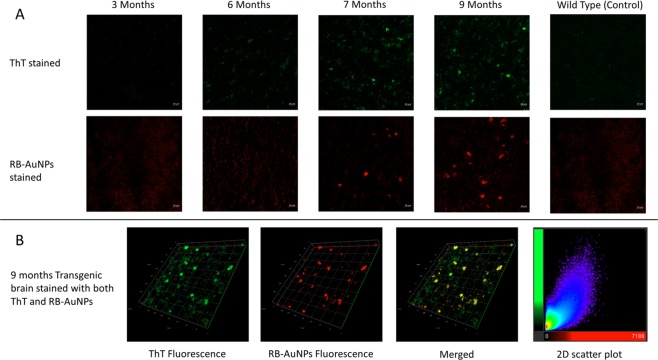
Confocal imaging of transgenic mouse brain slices. (**A**) Confocal images of transgenic mice brain slices in different ages, the brain slices were stained separately using ThT or RB-AuNPs. Brain slices from 9 months old wild type mice were used as control groups. (**B**) Confocal z-stack images of transgenic mice brain slice stained with both ThT and RB-AuNPs. The 2d scatter histogram was plotted by setting the intensities of RB-AuNPs as x-axis and the intensities of ThT as y-axis.

The transgenic mouse brain slices used in this study is a generic humanized animal model for AD research. This model was developed by Oddo *et al*. in 2003^51^. The 3xTg mice show age-dependent amyloidosis progress. Previous studies have detected apparent extracellular amyloid plaques in the mice’s frontal cortex after 6 months old^51,52^. Figure 10(A) shows the confocal images of mouse brains stained separately with ThT or RB-AuNPs. For both types of stained brain slices, only weak emissions were detected in the control and young-aged (3 and 6 months) brains. At 7 months old, plaques become visible, and more plaques were detected in 9 months old brains. Figure 10(B) shows the z-stack images of 9 months old brain slices stained with both ThT and RB-AuNPs. The 2D scatter plot of the fluorescence from ThT and RB-AuNPs shows a partially co-localization relationship with a Pearson’s correlation coefficient (PCC) equals to 0.6403. It needs to be pointed out that the RB-AuNPs fluorescence has a higher degree of co-occurrence: the Manders coefficient of RB-AuNPs fluorescence MC_R_ = 93.62%. A Manders coefficient of 93.62% means that 93.62% of the RB-AuNPs fluorescence coincided with the ThT fluorescence. On the other hand, a considerable amount of the ThT fluorescence was detected without accompanying RB-AuNPs fluorescence: The Manders coefficient of ThT fluorescence MC_T_ = 46.56%. The difference in the co-localization degree is due to the non-specific staining of ThT dyes, which lead to a lower signal-to-noise ratio.

## Discussion

In this paper, RB conjugated gold nanoparticles are prepared and used as bifunctional nanoprobe for SERS-based Aβ42 peptides detection and fluorescence imaging of amyloid plaques. It is demonstrated that the Raman scattering of RB molecules is significantly enhanced when conjugated to the AuNPs and the enhanced Raman spectrum found to exhibit detectable changes in the presence of amyloid peptides. In addition, it is also inferred that the interaction between Aβ42 peptides and RB-AuNPs generate strong enhanced fluorescence, which was sufficient for the labeling of amyloid plaques in mouse brain slices.

Fluorescence imaging using the RB-AuNPs revealed an age-dependent amyloid deposition, consistent with reported previous studies as detailed in the discussion section^52^. The labelling specificity is also comparable with the commonly used amyloid dye. Based on the various observations and illustrations made through this manuscript, it can be concluded that a proof-of-concept study for the application of multifunctional nanoprobes is successfully carried out for the detection of amyloid-based on amyloid biomarking, which leads to the early diagnosis of AD. Based on this study, it is further envisaged that RB-AuNPs nanoparticles, which are successfully applied as inhibitors for amyloid aggregation (Supplementary Fig. S7), hold great potential to be utilized as theranostic agents for amyloidosis in the near future.

## Methods

### Materials

Aβ42 peptides and supplementary 1% NH_4_OH were obtained from Anaspec Inc. (Fremont, USA). Amine functionalized gold nanoparticles were purchased from Cytodiagnostics Inc. (Ontario, Canada). Rose Bengal disodium salt, 2-(N-morpholino)ethanesulfonic acid (MES), Paraformaldehyde (PFA), Thioflavin T (ThT), 1-ethyl-3-[3-dimethylaminopropyl] carbodiimide hydrochloride (EDC) and N-hydroxysuccinimide (NHS) were purchased from Sigma-Aldrich Co. LLC. FluorSave^®^ mounting medium was purchased from EMD Millipore. Disposable PD-10 Desalting column was purchased from GE Healthcare (Sweden). MTT Cell Viability Assay Kit was provided by Biotium, Inc (Fremont, USA).

### Preparation of RB-AuNP complex

The conjugation of RB to amine-functionalized AuNPs was conducted through a two-step EDC/NHS reaction. First, 10 μl of RB stock solution (10 mM in water) was diluted with 500 μl of 0.1 M MES buffer (PH = 5.0). Then 4 mg EDC and 6 mg NHS were added to the reaction vial. The mixture was gently stirred at room temperature for 20 mins at RT, protected from light. After the activation, 200 μl of AuNPs stock solution (2.46 mg/ml) was mixed with the RB solution. The mixture was stirred for another 3 hours at RT, protected from light. After the conjugation, the sample was centrifuged at 13k RPM for 10 mins and the supernatant was removed. The centrifugation was repeated for two more times using DI water to remove un-conjugated RB. The final RB-AuNP pellet was weighted and re-dispersed in DI water at a mass concentration of 0.3 mg/ml.

### Characterization

Transmission electron microscope (TEM) was conducted using JEM-2010 electron microscope (JEOL Ltd, Japan) under 200 kv voltage. The absorption spectra were measured using Shimadzu UV-1800 UV-VIS spectrophotometer. The fluorescent emission was recorded using FS5 Spectrofluorometer (Edinburgh Instruments, UK). The Raman spectrum was measured using inVia^TM^ Basis confocal Raman microscope (Renishaw plc, UK). The Zeta potential and DLS measurements were conducted using Zeta Nanosizer (Malvern Instruments, UK).

### Cytotoxicity assay

5 × 10^3^ per well of HEK-293 cells were seeded into a 96-well plate with 100 μl of DMEM/F-12 medium (10% FBS). The 96-well plate was incubated overnight at 37 °C with 5% CO_2_. The medium was refreshed with 100 μl of fresh medium containing PBS or various concentrations of RB-AuNP. The treated cells were incubated for 24 hours and then refreshed with 100 μl fresh medium. Next, 10 uL of MTT solution was added to each well and incubated at 37 °C for another 4 hours. After that, 200 μl of DMSO was added to each well. The 96-well plate was covered with tin foil and gently shakes at room temperature for 30 mins to fully dissolve formazan salt. The absorbance of each well was measured at 570 nm using Synergy^TM^ H1 microplate reader (BioTek, USA). Background absorbance was measured at 630 nm. Each sample was tested in 3 parallel wells.

### Preparation of Aβ42 peptides solution

The oligomeric Aβ42 peptides stock solution was prepared by NaOH treatment, following a previous study^53^ with minor modification. Briefly, 1 mg of lyophilized Aβ42 peptides was dissolved in 1 ml of ice-cold NaOH solution (0.01 M). The vial was then sonicated for 1 min to fully dissolve the peptides. The actual peptides concentration was measured to be 175 μM by UV absorbance with an extinction coefficient of 1450 cm^−1^M^−1^ at 280 nm^54^. The solution was separated into multiple aliquots of 100 μl and stored at −20 °C.

### Fluorescent detection of Aβ42 peptides & RB-AuNPs

Interaction To confirm the interaction between Aβ42 peptides and the as-prepared RB-AuNPs, different concentrations of Aβ42 peptides were mixed with 1 μg/ml of RB-AuNPs. For comparison purpose, same concentrations of Aβ42 peptides were also mixed with 1 μM of free RB. The mixture was transferred to a 96-well plate and the fluorescence emission was recorded (560/590 nm ex/em) using Synergy H1 Multi-Mode plate reader (BioTek Instrument Inc.).

To explore the competing effect between ThT and RB-AuNPs upon Aβ42 peptides binding. 5 μM of Aβ42 peptides solutions were mixed with 40 μM of ThT solutions. The ThT fluorescence was measured at 440/480 nm ex/em. Then different concentrations of RB-AuNPs were added to the ThT/Aβ42 mixture, and then the ThT fluorescence was measured again. To remove the effect of fluorophore crosstalk, Mixed ThT/RB-AuNPs solutions without Aβ42 peptides were used as blank samples. The viability was then calculated using Origin 9.4.

### SERS Detection of Aβ42 peptides

Different concentration of Aβ42 peptides was mixed with 1 μg/ml of RB-AuNPs. The concertation of RB-AuNPs is chosen based on the signal intensity (Supplementary Fig. S4). The mixture was dropped on the surface of pre-cleaned glass slides and left unperturbed for more than 6 hours to dry. The Raman spectra of dried samples were measured using a 633 nm laser (30 mW laser power, 10% laser intensity, 3 accumulations, 10 s exposure time) and 1800 grooves/cm grating.

### Confocal imaging of transgenic mouse brain slices

Mouse brain slices (15 μm thickness) were harvested from different aged transgenic mice. Transgenic mice were engineered with three mutations (PP Swedish, MAPT P301L, and PSEN1 M146V) according to a previous study^51^. The brain slices were first incubated with 4% PFA solution for 5 mins in order to fix the brain tissue. After the fixation, the PFA was washed away using PBS. Then the brain slices were stained by dropping 50 μl of RB-AuNPs solution (10 μg/ml) or ThT solution (40 μM) on the surface of the brain slices. After 10 mins incubation, the brain slices were washed twice with PBS to remove excess staining agents. Pre-cleaned coverslips were mounted on top of the brain slices using FluorSave mounting medium. The confocal images were obtained using an LSM 800 Inverted Confocal Microscope (Zeiss, German). The co-localization analysis was conducted using Imaris 9.1 software (Bitplane, Switzerland). Pearson’s correlation coefficient (PCC) and Manders coefficient of the respective signals were calculated to quantify the degree of co-localization. The PCC is a coefficient that quantifies the linear correlation between two variables. It is calculated by dividing the covariance of the two variables by the product of their standard deviations. The value of PCC ranges from −1 to +1. Two variables are total positively correlated when PCC is +1 and totally negatively correlated when PCC is −1. The Manders coefficient measures the coincidence of signals from one channel with respect to signals from another channel. It is calculated by dividing the total intensities of coincided pixels by the total intensities of all pixels^55^.

Mice used in this study were raised and harvested in accordance with relevant guidelines and regulations. All protocols are approved by the Institutional Animal Care and Use Committee (IACUC)-NTU (Ref. No. - LKCMedicine, NTU-151045).

## Supplementary information

Bifunctional Fluorescent/Raman Nanoprobe for the Early Detection of Amyloid
