# Supplementary material for: Bifunctional Fluorescent/Raman Nanoprobe for the Early Detection of Amyloid
Source: Sci Rep. 2019 Jun 11;9:8497. doi: 10.1038/s41598-019-43288-2 (PMC6560097; doi:10.1038/s41598-019-43288-2)
Supplement: Supplementary file 1 — Bifunctional Fluorescent/Raman Nanoprobe for the Early Detection of Amyloid [file 41598_2019_43288_MOESM1_ESM.docx]

Supporting Information

**Bifunctional Fluorescent/Raman Nanoprobe for the Early Detection of Amyloid**

**Yang Xia^1,2^, Parasuraman Padmanabhan^2,*^, Sreelatha Sarangapani1^1^, Balázs Gulyás^2^, Murukeshan Vadakke Matham^1,*^**

1School of Mechanical and Aerospace Engineering, Center for Optical and Laser Engineering (COLE), Nanyang Technological University (NTU), Singapore 639798

2Lee Kong Chian School of Medicine, Nanyang Technological University (NTU), Singapore 637553

**Differential light scattering (DLS) measurement of AuNPs**

DLS measurements of the AuNPs were conducted before and after the conjugation. As shown in Figure S1, the hydrodynamic size of the AuNPs increased slightly (from 29.10 nm to 32.31 nm). Considering the effect of conjugated RB molecules, such a small change in the hydrodynamic size suggests that only a slight aggregation has occurred.


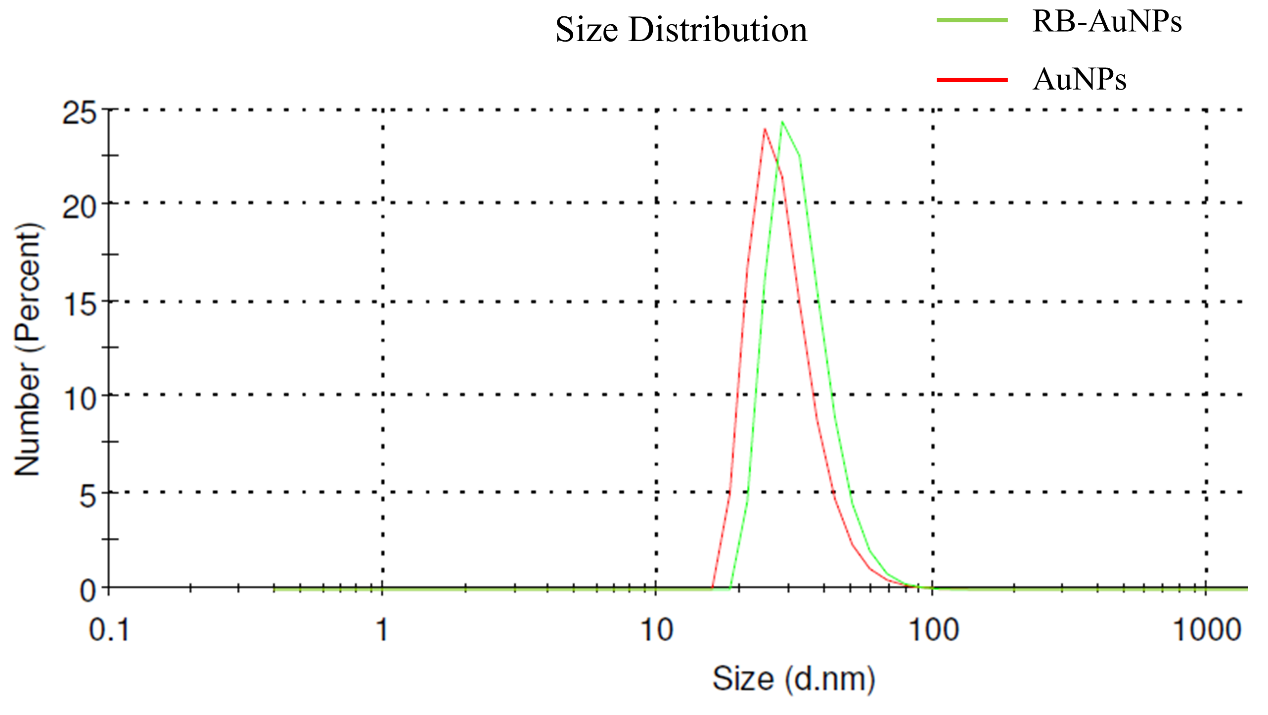
Figure S1. hydrodynamic size of the AuNPs and RB-AuNPs

**Colloidal stability of RB-AuNPs in the cell medium**

The colloidal stability of RB-AuNPs in the cell medium was investigated by comparing the UV-Vis spectra of RB-AuNPs in PBS buffer and the cell culture medium. It has been reported that the aggregation of gold nanoparticles would lead to a red shift in the absorption spectra since the aggregation can shorten the distance between particles [^1^](#_ENREF_1). As show in the following figure, a minor red shift in the UV-Vis spectra of RB-AuNPs was observed upon the transfer of RB-AuNPs from the stock PBS buffer to the cell culture medium (DMEM/F12). This observation indicates a slight aggregation of RB-AuNPs particles.


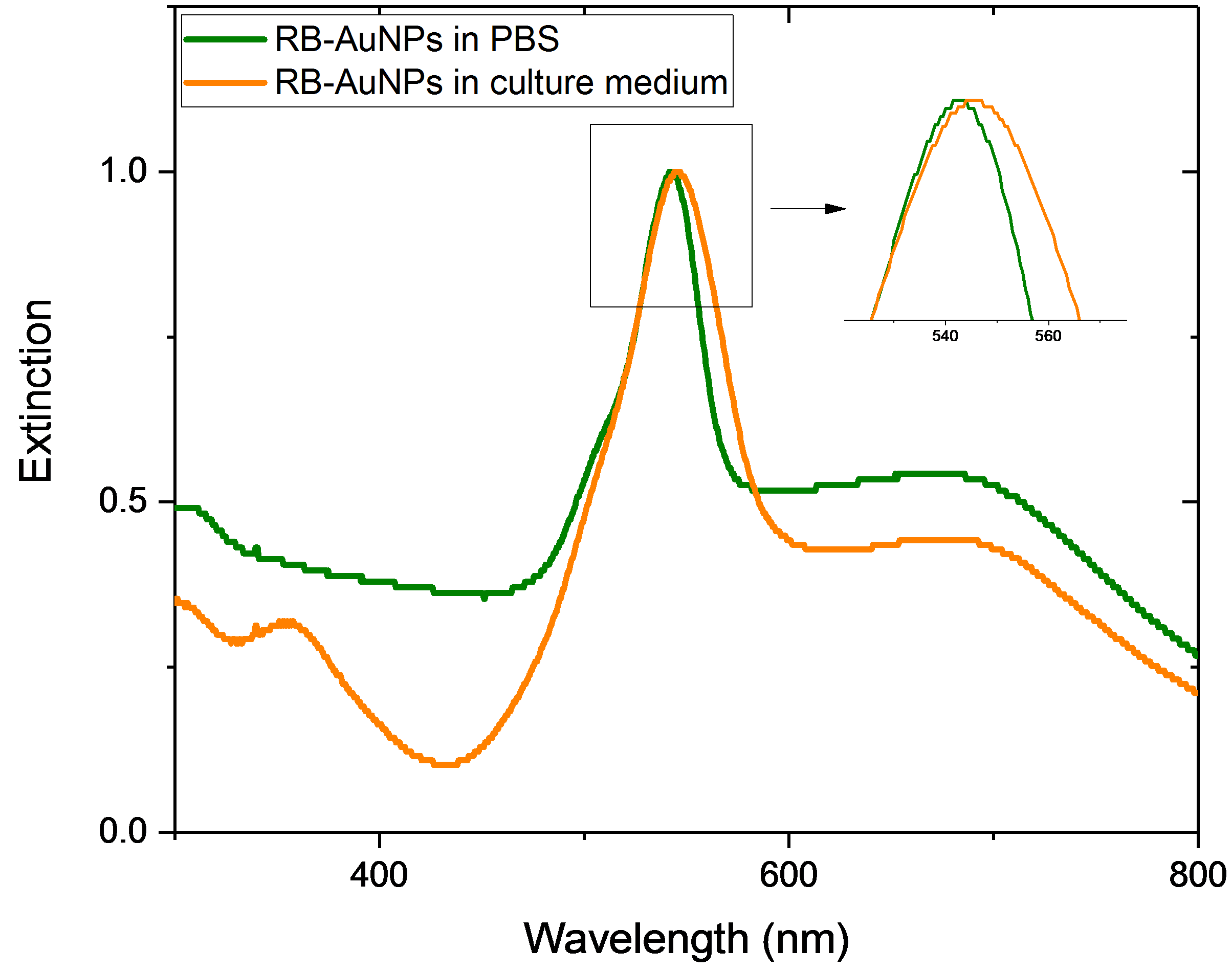


Figure S2. UV-VIS spectra of RB-AuNPs in PBS and cell culture medium.

**Biocompatibility of the RB-AuNPs**

Apart from the cell proliferation assay, the morphology of cells is another important indicator of the biocompatibility of nanoparticles[^2^](#_ENREF_2). Figure S3 shows the confocal images of HEK-293 cells after 24 hours incubation in the presence or absence of RB-AuNPs. After the incubation, the HEK-293 cells were also stained with DAPI and then fixed with 4% paraformaldehyde solution. The treated cells showed no detectable changes in the cell morphology compared with the control cells. The shapes of cell nuclei also remain intact. These observations further prove the excellent biocompatibility of the RB-AuNPs.


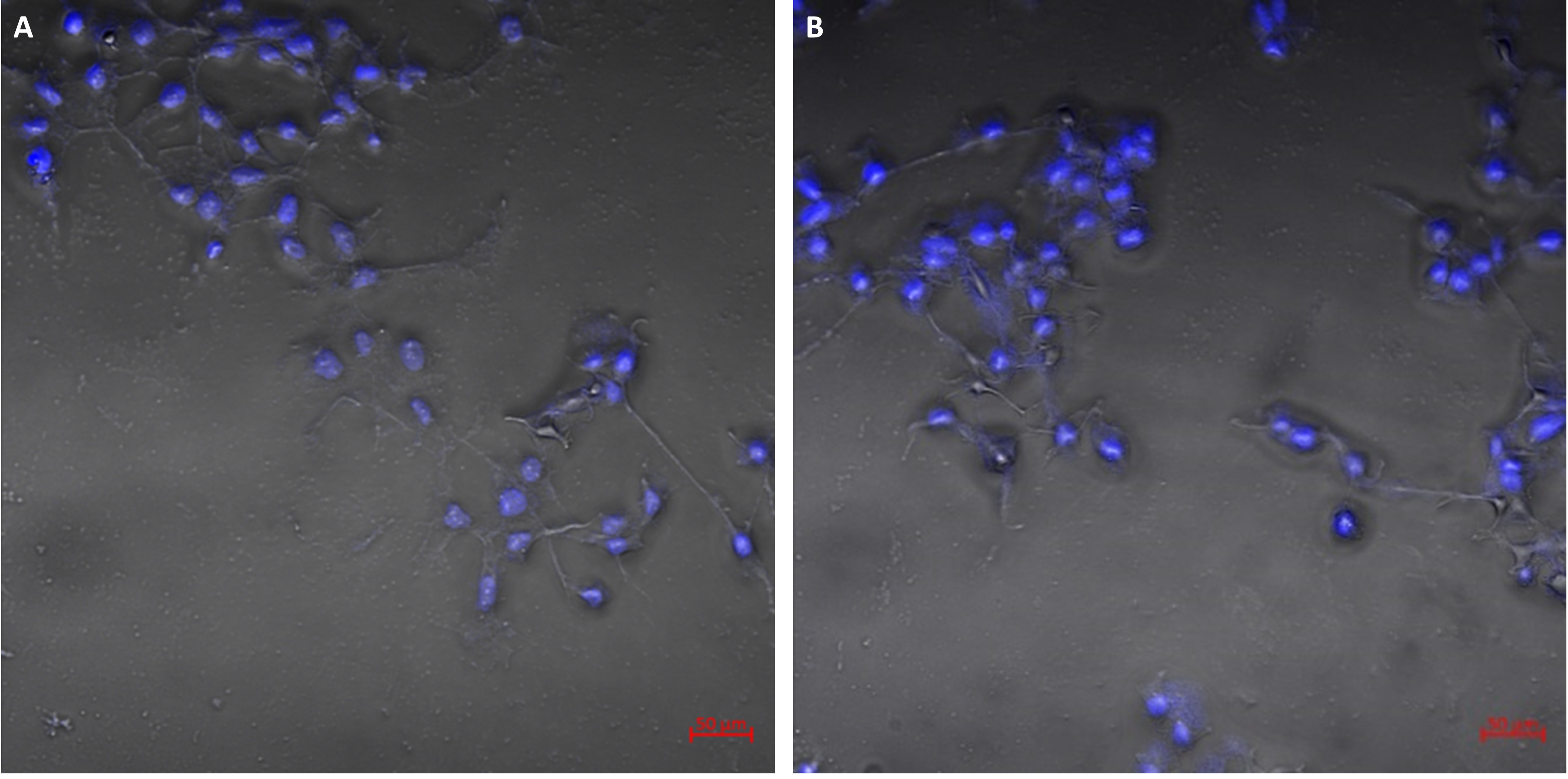


Figure S3. Confocal images of HEK-293 cells after 24 hours incubation in the presence or absence of RB-AuNPs: (a) control and (b) incubated with 20 μg/ml of RB-AuNPs. The cells nuclei were stained with DAPI (blue).

**Concentration effect of RB-AuNPs**

Figure S4 shows the concentration effect of RB-AuNPs. In order to find the suitable concentration for the Raman detection, we mixed different concentrations of RB-AuNPs with 2 μM Aβ42. The spectra show that the spectra features are obvious enough when the RB-AuNPs’ concentration is greater than 1 μg/ml, considering the potential toxic effect of RB molecules, the actual concentration used for the SERS detection of Aβ42 was decided to be 1 μg/ml.


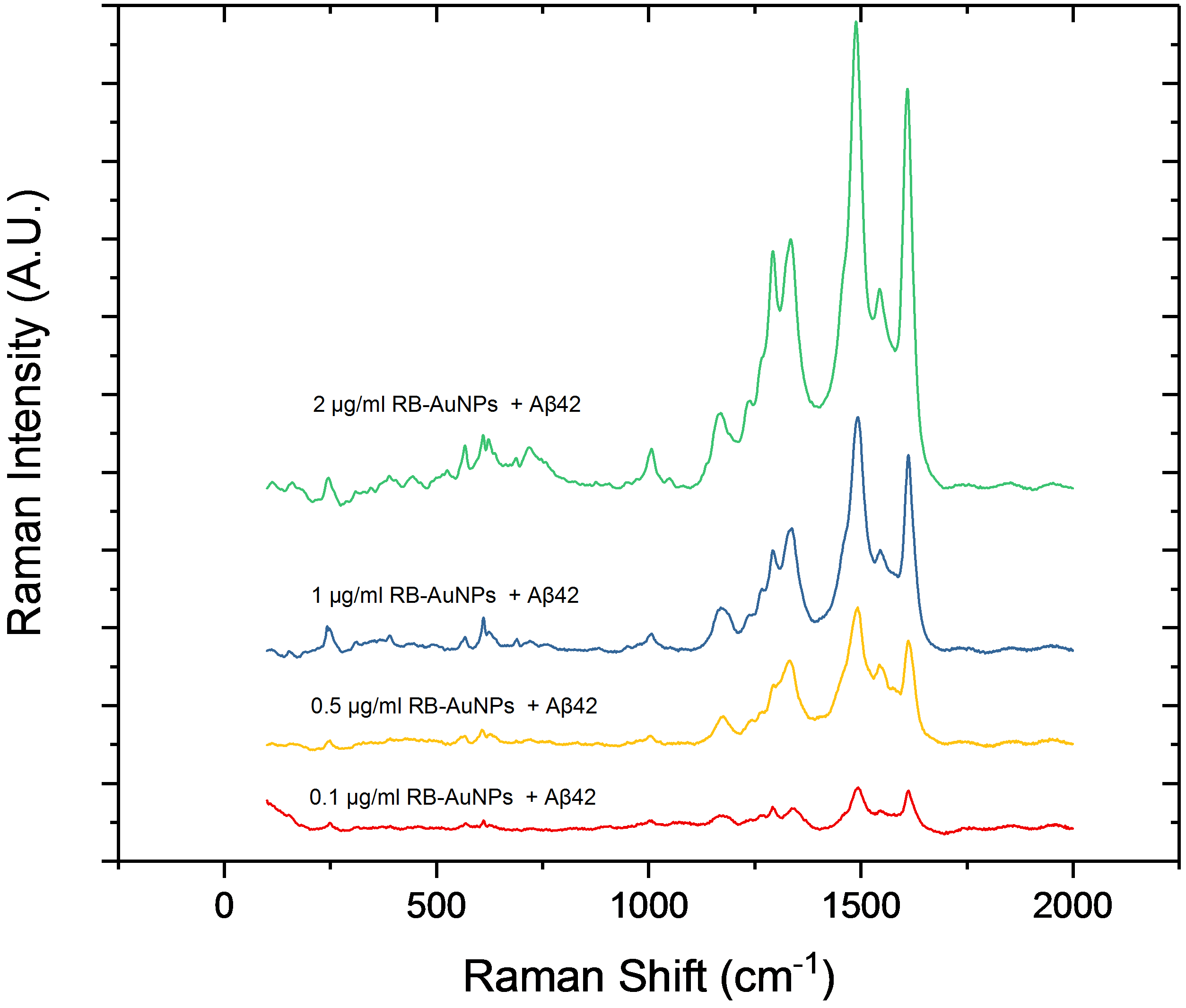


Figure S4. Concentration effect of RB-AuNPs when incubated with 2 μM Aβ42.

**Selectivity towards amyloid peptides**

The amyloid-selectivity of RB-AuNPs was tested by mixing the nanoprobe with 2 μM Lipocalin prostaglandin D synthase (L-PGDS). L-PGDS is an enzyme that regulates the synthesis of a signaling mediator prostaglandin D_2_ [^3^](#_ENREF_3). L-PGDS are mainly presented in the cerebrospinal fluid (CSF) [^4^](#_ENREF_4). Considering that CSF is also an important source for Aβ42 peptides [^5^](#_ENREF_5), we can say that L-PGDS coexists with Aβ42 peptides. As shown in the following figure, the Raman spectrum of RB-AuNPs shows only small changes. The ratio between the two major peaks (intensity at 1490 cm^-1^/intensity at 1610 cm^-1^) changed from 0.780 to 0.769. This result suggests that the changes in the ratio of major peaks are specifically associated with amyloid peptides.


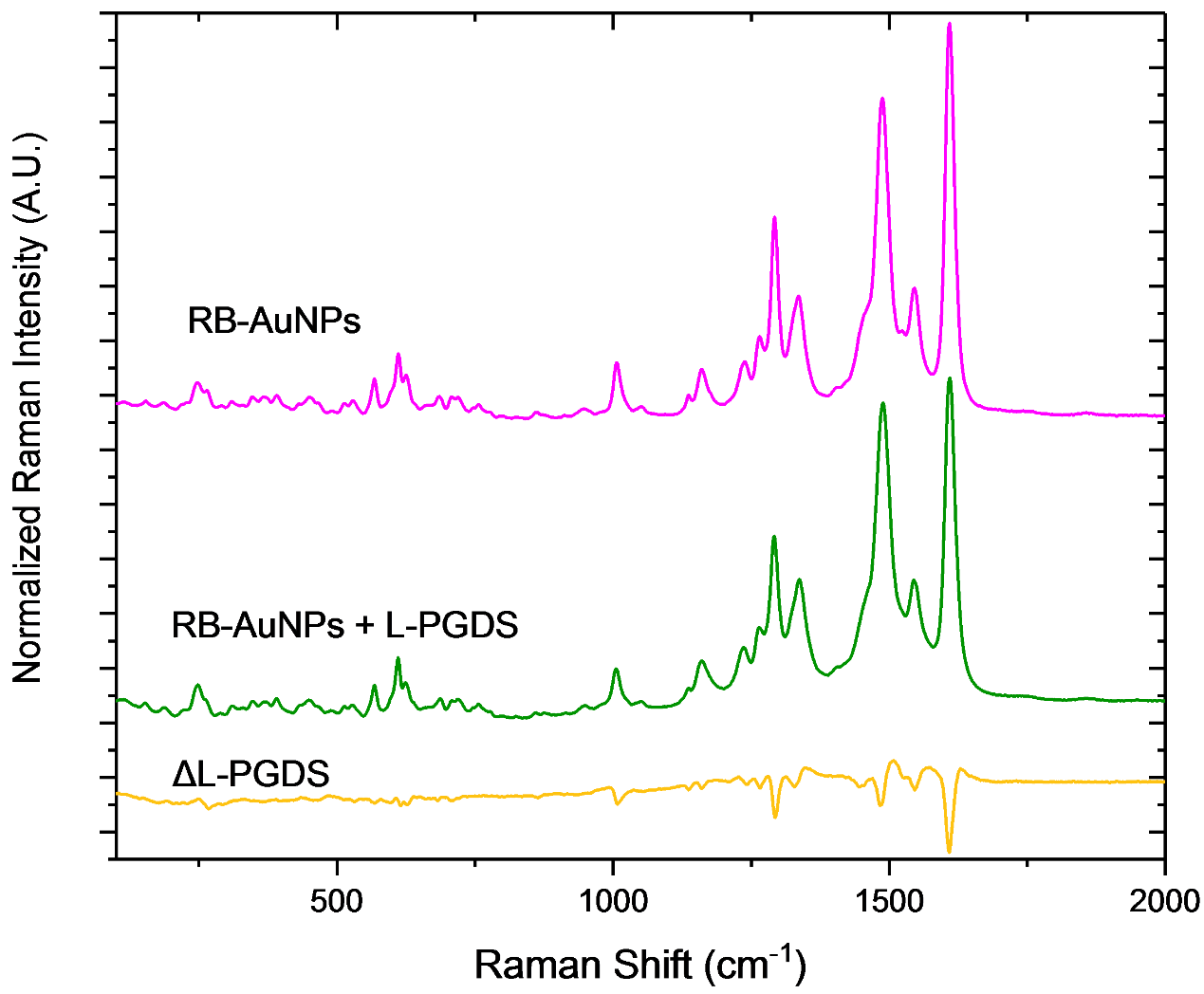


Figure S5. SERS spectrum of RB-AuNPs in presence or absence of L-PGDS. The orange curve is obtained by digital subtraction of the (RB-AuNPs) spectrum from the (RB-AuNPs + L-PGDS) spectrum. The ratio between the two major peaks (intensity at 1490 cm^-1^/intensity at 1610 cm^-1^) changed from 0.780 to 0.769.

**Raman spectral mapping of mouse brain slices**

Raman mapping of mouse brain slices were conducted using inVia^TM^ Basis confocal Raman microscope. The brain slices were stained by dropping 50 μl of RB-AuNPs solution (10 μg/ml) on the surface of the brain slices. After 10 mins incubation, the brain slices were washed twice with PBS to remove excess staining agents. The Raman imaging was performed using 20X objective (Leica) with a 633 nm laser (30 mW laser power, 10% laser intensity, 1s exposure time). For each sample, 2700 measurement spots were taken and the size of each spot is 2.5 X 2.5 μm^2^. After the measurement, the Raman mapping was generated based on signal-to-baseline algorithm using WiRE^TM^ 3.2 software. Two spectral ranges were selected to generate the Raman mapping were plotted at two spectral ranges which covers the two major peaks in the RB-AuNPs’ Raman spectrum: 1470 to 1510 cm^-1^ , covers the peak at 1490 cm^-1^, and 1590 to 1630 cm^-1^, covers the peak at 1610 cm^-1^. As we can see from figure S6, the Raman images generated from both ranges did not show significant differences between the control (wild type mouse brain) and the experimental (transgenic mouse brain) groups. However, by highlighting the spots in which R_peak_ (R_peak_= Intensity at 1490 divided by the intensity at 1610 cm^-1^) is greater than 1, an obvious difference was observed. The significantly increased density of highlighted spots indicated an increased amyloid deposition in the transgenic mouse brain.


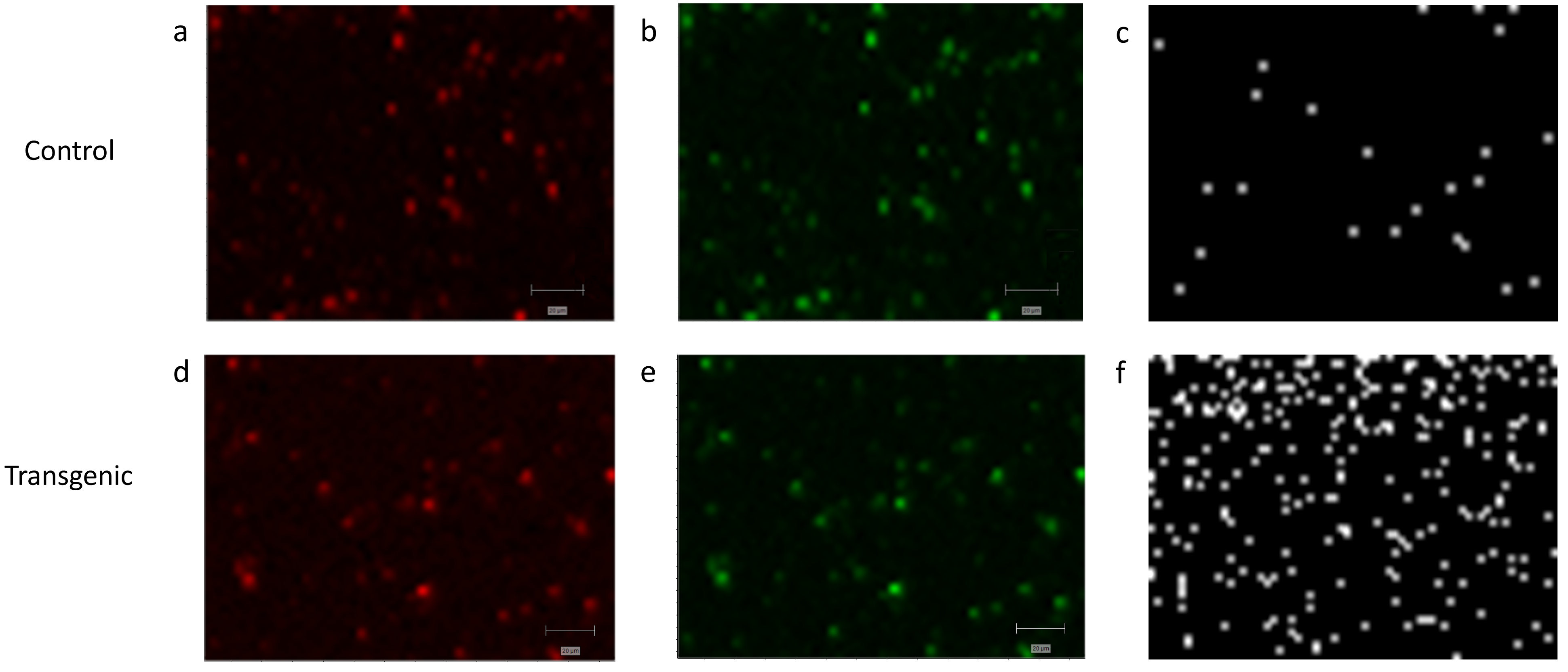


Figure S6. Raman mapping of mouse brain slices stained with RB-AuNPs. (a) & (d) Mappings plotted by calculating the signal to baseline area at the range of 1470 to 1510 cm^-1^ for the control group (9-month wild-type mouse) and experimental group (9-month transgenic mouse). (b) & (e) Mappings plotted by calculating the signal to baseline area at the range of 1590 to 1630 cm^-1^ (signal to baseline area). (c) & (f) highlight the spots in which the R_peak_ (Intensity at 1490 divided by the intensity at 1610 cm^-1^) value is great than 1.

**Effect of RB-AuNPs on the fibrilization of Aβ42 peptides.**

The presence of nanoparticles has been found capable to affect the fibrilization process of amyloid peptides, and this phenomenon are considered as a proof for the particles-peptides interaction[^6-7^](#_ENREF_6). The effect of RB-AuNPs on the Aβ42 fibrilization was investigated by incubating 10 μM Aβ42 with 10 μg/ml RB-AuNPs at 37 °C. After 6 hours of incubation, the morphology of the resultant amyloid aggregates was characterized by TEM. As show in figure S7, when incubated with RB-AuNPs, the Aβ42 aggregation process (b) was clearly inhibited compared to the control group (a). This changed aggregation indicated the strong affinity between RB-AuNPs and Aβ42 peptides, which reduced the quantity of available Aβ42 oligomer for self fibrilization.


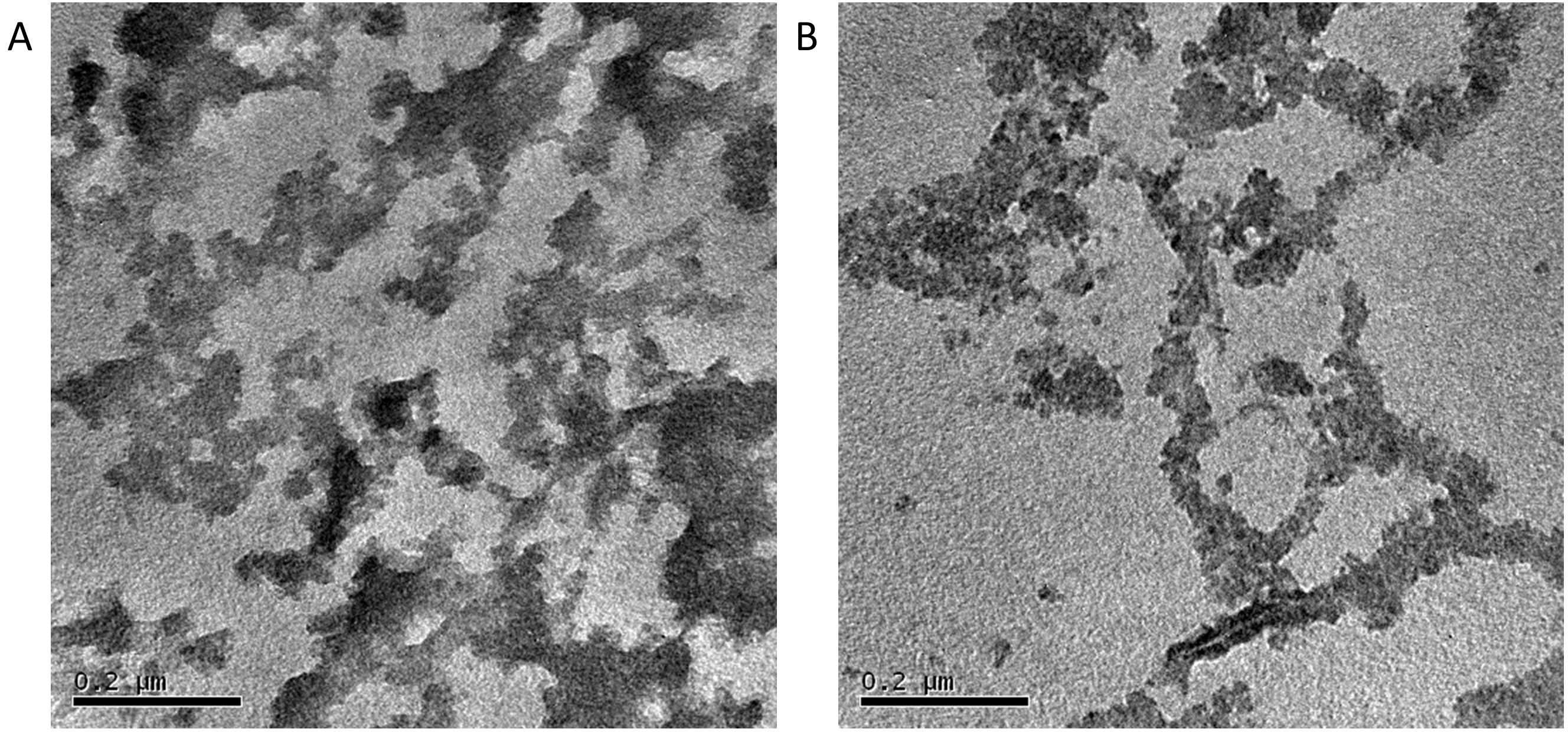


Figure S7. The morphology of 10 μM Aβ42 aggregates after 6h incubation (37 °C) in the absence (a) or presence of 10 μg/ml RB-AuNPs. The fibrilization process was altered by the presence of nanoparticles, indicated the affinity between Aβ42 and RB-AuNPs.)

**Reference**

1. Gao, J.; Huang, X.; Liu, H.; Zan, F.; Ren, J., Colloidal stability of gold nanoparticles modified with thiol compounds: bioconjugation and application in cancer cell imaging. *Langmuir* **2012,** *28* (9), 4464-4471.

2. Wang, S.; Li, K.; Chen, Y.; Chen, H.; Ma, M.; Feng, J.; Zhao, Q.; Shi, J., Biocompatible PEGylated MoS2 nanosheets: Controllable bottom-up synthesis and highly efficient photothermal regression of tumor. *Biomaterials* **2015,** *39*, 206-217.

3. Lim, S. M.; Chen, D.; Teo, H.; Roos, A.; Jansson, A. E.; Nyman, T.; Trésaugues, L.; Pervushin, K.; Nordlund, P., Structural and dynamic insights into substrate binding and catalysis of human lipocalin prostaglandin D synthase. *Journal of lipid research* **2013**, jlr. M035410.

4. Watanabe, K.; Urade, Y.; Mader, M.; Murphy, C.; Hayaishi, O., Identification of β-trace as prostaglandin D synthase. *Biochemical and biophysical research communications* **1994,** *203* (2), 1110-1116.

5. Strozyk, D.; Blennow, K.; White, L.; Launer, L., CSF Aβ 42 levels correlate with amyloid-neuropathology in a population-based autopsy study. *Neurology* **2003,** *60* (4), 652-656.

6. Liao, Y. H.; Chang, Y. J.; Yoshiike, Y.; Chang, Y. C.; Chen, Y. R., Negatively Charged Gold Nanoparticles Inhibit Alzheimer's Amyloid‐β Fibrillization, Induce Fibril Dissociation, and Mitigate Neurotoxicity. *Small* **2012,** *8* (23), 3631-3639.

7. Wang, M.; Kakinen, A.; Pilkington, E. H.; Davis, T. P.; Ke, P. C., Differential effects of silver and iron oxide nanoparticles on IAPP amyloid aggregation. *Biomaterials science* **2017,** *5* (3), 485-493.
